# Supplementary material for: Global vegetation resilience linked to water availability and variability
Source: Nat Commun. 2023 Jan 30;14:498. doi: 10.1038/s41467-023-36207-7 (PMC9886942; doi:10.1038/s41467-023-36207-7)
Supplement: Supplementary file 1 — Supplementary Information [file 41467_2023_36207_MOESM1_ESM.pdf]

Supplementary Information File: Global Vegetation Resilience Linked to Water Availability and Variability

Taylor Smith[1] and Niklas Boers[2,3,4]
[1]Institute of Geosciences, Universität Potsdam, Germany
[2]Earth System Modelling, School of Engineering & Design, Technical University of Munich, Germany
[3]Potsdam Institute for Climate Impact Research, Germany
[4]Department of Mathematics and Global Systems Institute, University of Exeter, UK

Corresponding author:
Taylor Smith
Email: tasmith@uni-potsdam.de

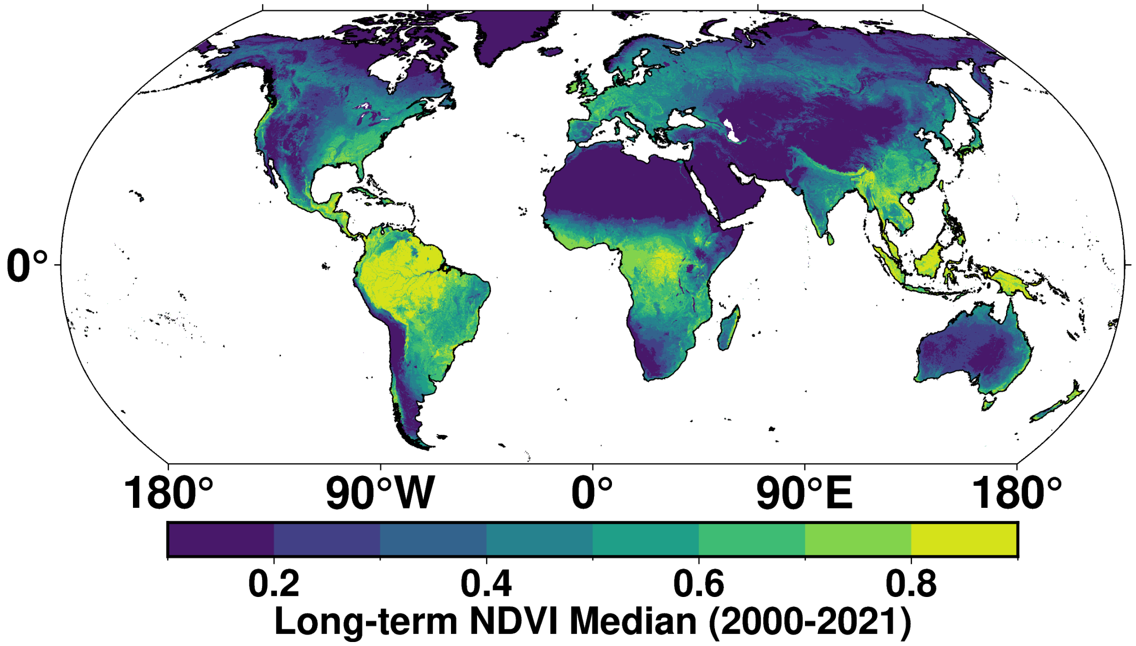

Figure S1: Long-term (2000-2021) median normalized difference vegetation index (NDVI) from MODIS MOD13.

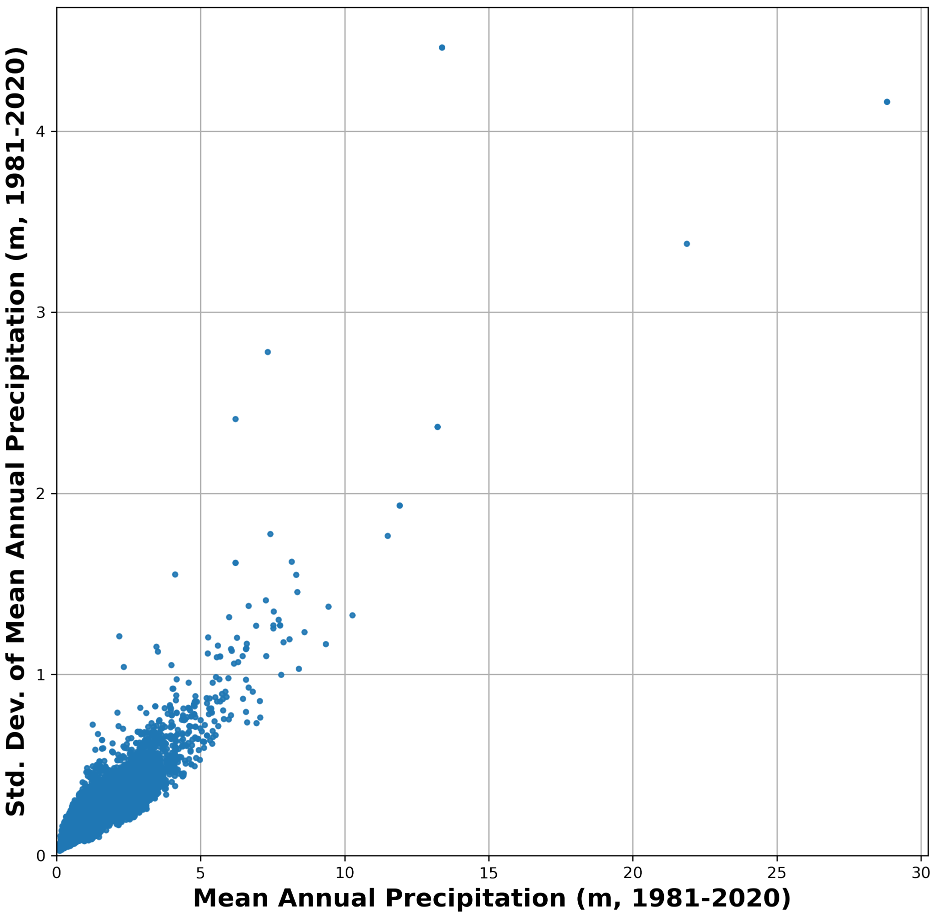

Figure S2: Relationship between mean annual precipitation and mean annual precipitation standard deviation for all points. There is a strong linear dependence between precipitation variability and absolute amount.

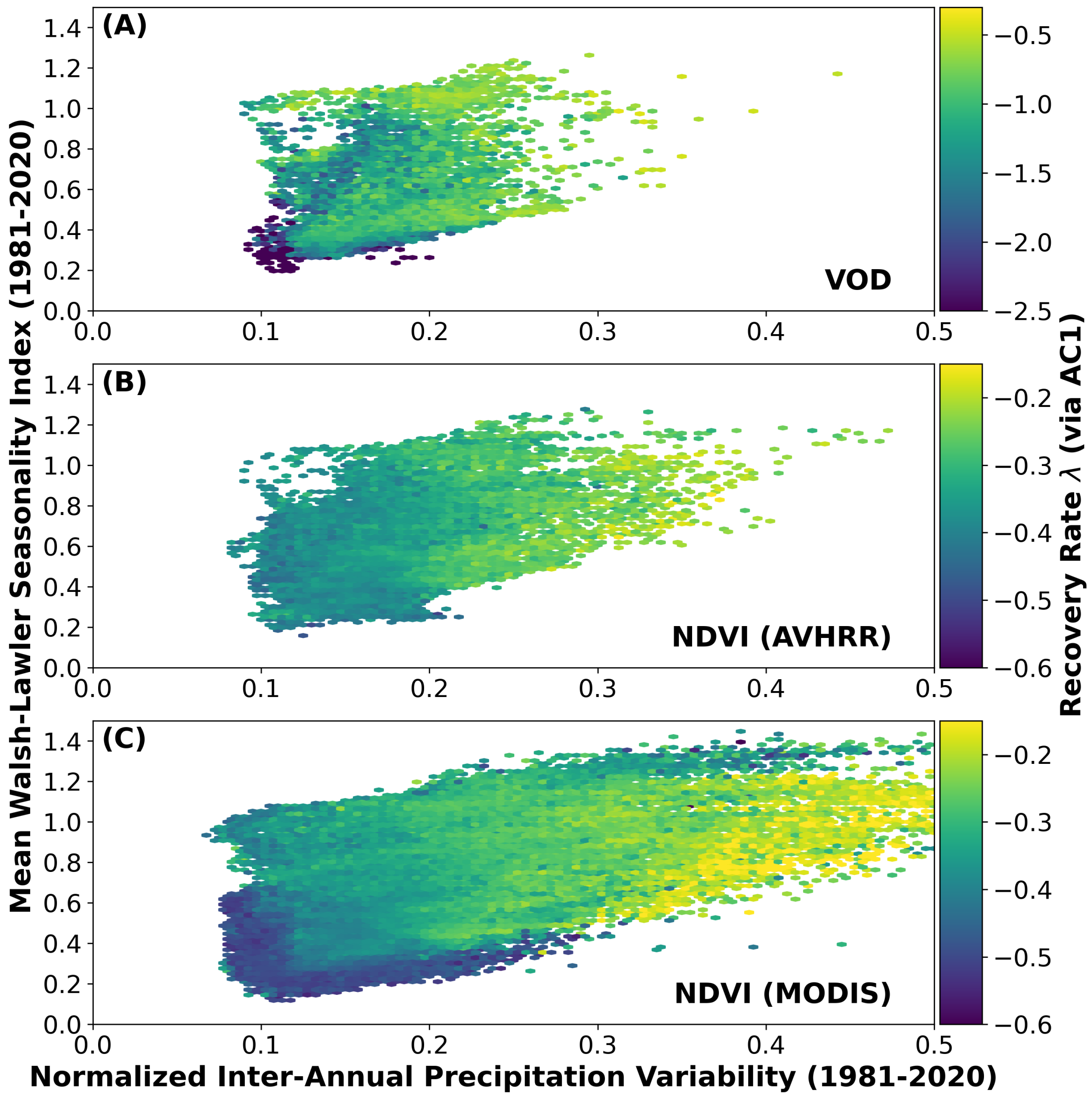

Figure S3: Relative importance of intra- and inter-annual precipitation variability in controlling vegetation resilience. (A) vegetation optical depth (VOD), (B) GIMMS3g normalized difference vegetation index (NDVI), and (C) MODIS NDVI. Hexbins colored by recovery rate computed from AC1 (minimum five points per bin).

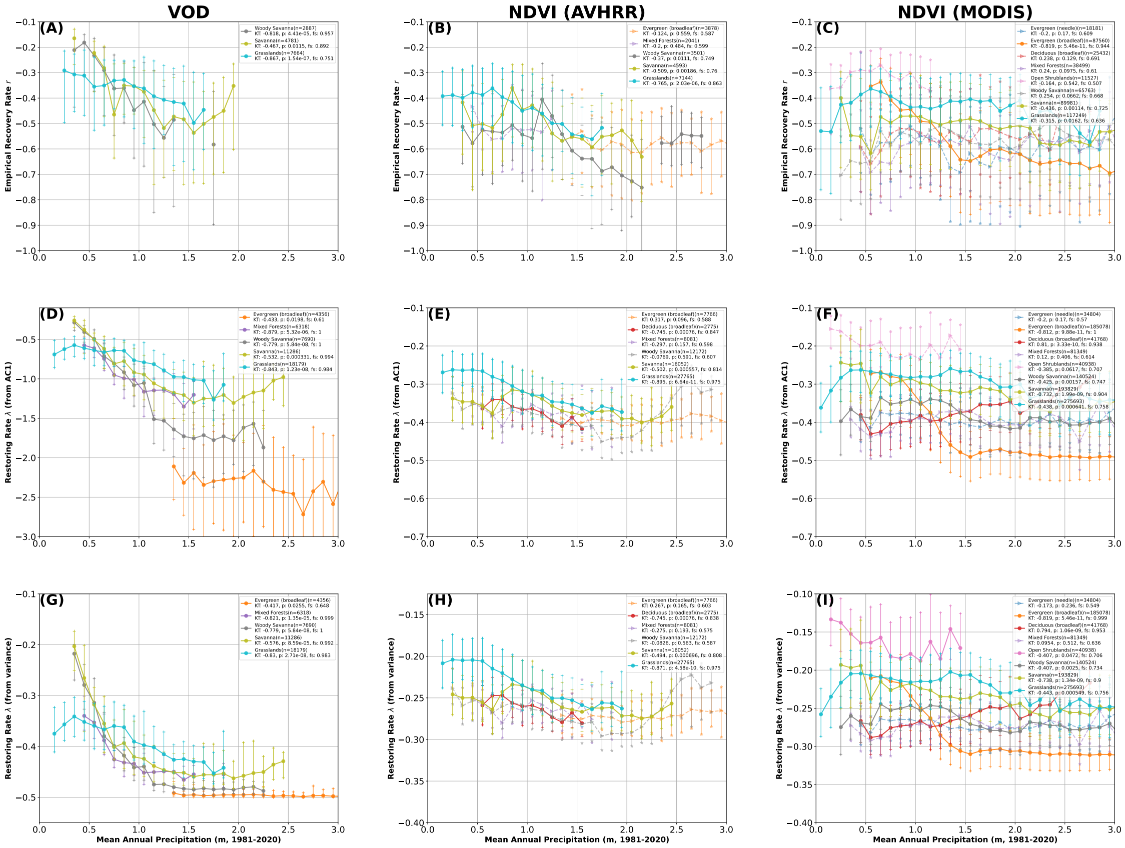

Figure S4: Vegetation resilience as a function of mean annual precipitation (1981-2020, ERA) at the global scale, separated by land cover type. Land covers with less than 1000 points or less than 10 bins of at least 50 members are omitted. Vegetation resilience estimated empirically (A,B,C), via the AC1 (D,E,F), and via the variance (G,H,I, Methods) for vegetation optical depth (VOD, left column), GIMMS3g normalized difference vegetation index (NDVI, middle column), and MODIS NDVI (right column). Binned medians shown as solid dots (p <0.05) and transparent arrows (p >0.05), with 25th to 75th percentiles of each bin shown as connected vertical lines capped with hatches. Kendall-Tau coefficients of the series of medians of each bin, their p-values, and the fraction of surrogate series consistent with the uncertainties that have the same sign as the median series (see Methods) are reported in the legend.

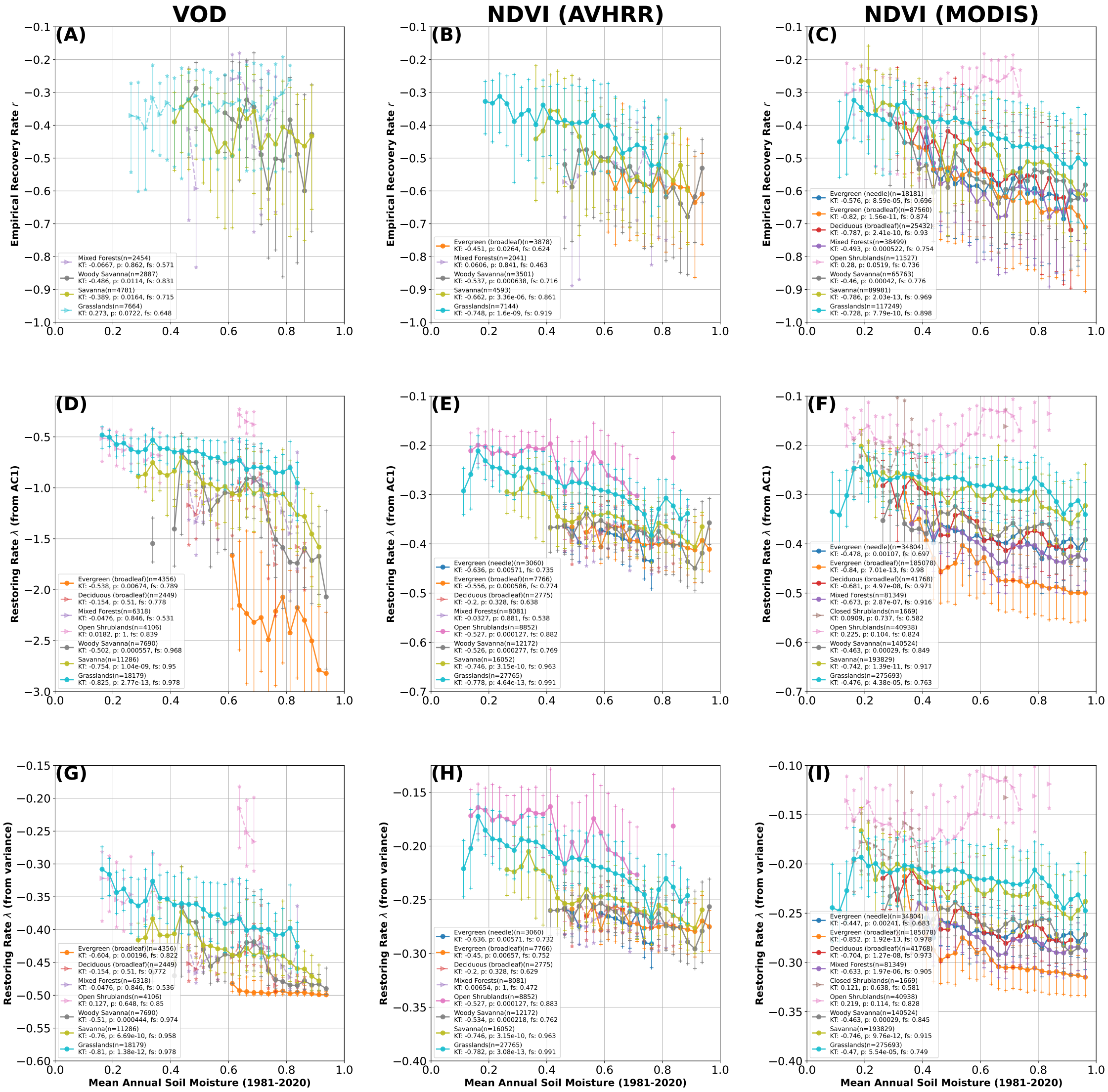

Figure S5: Vegetation resilience as a function of mean annual soil moisture (1981-2020, ERA) at the global scale, separated by land cover type. Land covers with less than 1000 points or less than 10 bins of at least 50 members are omitted. Vegetation resilience estimated empirically (A,B,C), via the AC1 (D,E,F), and via the variance (G,H,I, Methods) for vegetation optical depth (VOD, left column), GIMMS3g normalized difference vegetation index (NDVI, middle column), and MODIS NDVI (right column). Binned medians shown as solid dots (p <0.05) and transparent arrows (p >0.05), with 25th to 75th percentiles of each bin shown as connected vertical lines capped with hatches. Kendall-Tau coefficients of the series of medians of each bin, their p-values, and the fraction of surrogate series consistent with the uncertainties that have the same sign as the median series (see Methods) are reported in the legend.

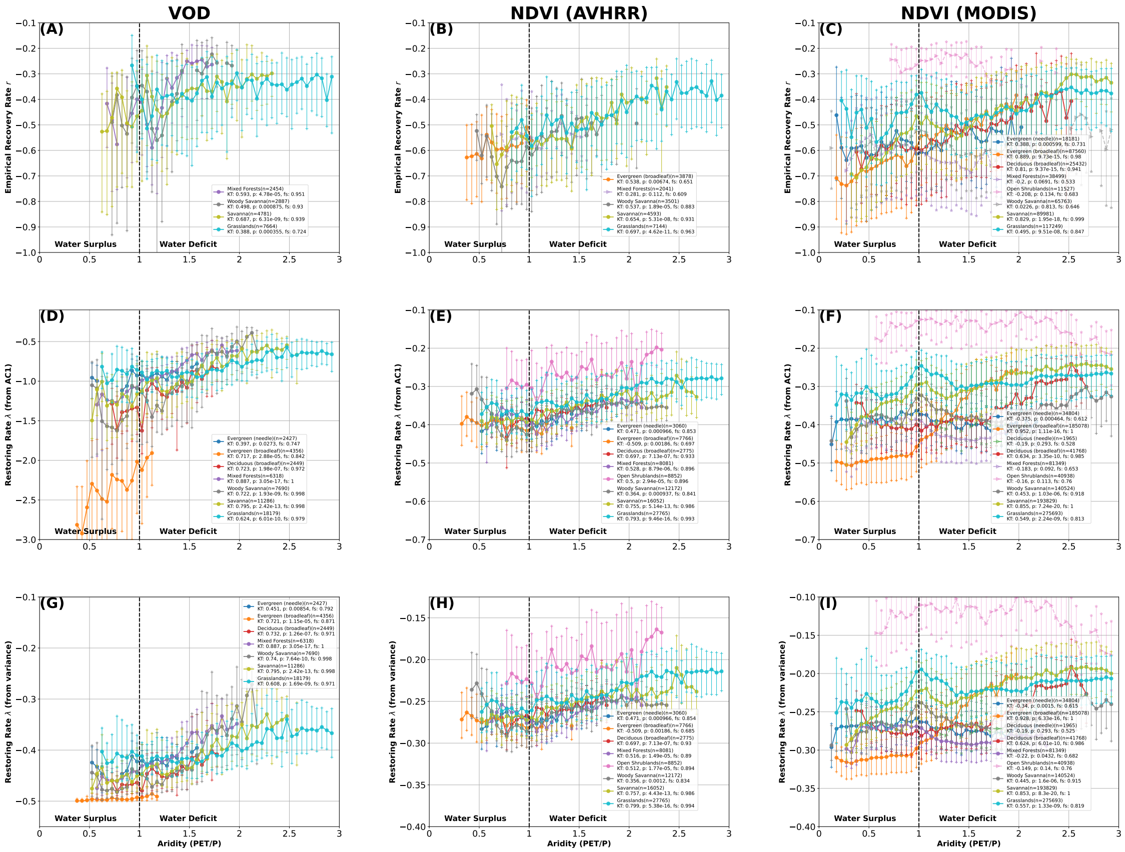

Figure S6: Vegetation resilience as a function of aridity at the global scale, separated by land cover type. Land covers with less than 1000 points or less than 10 bins of at least 50 members are omitted. Vegetation resilience estimated empirically (A,B,C), via the AC1 (D,E,F), and via the variance (G,H,I, Methods) for vegetation optical depth (VOD, left column), GIMMS3g normalized difference vegetation index (NDVI, middle column), and MODIS NDVI (right column). Binned medians shown as solid dots ($p<0.05$) and transparent arrows ($p>0.05$), with 25th to 75th percentiles of each bin shown as connected vertical lines capped with hatches. Kendall-Tau coefficients of the series of medians of each bin, their p-values, and the fraction of surrogate series consistent with the uncertainties that have the same sign as the median series (see Methods) are reported in the legend.

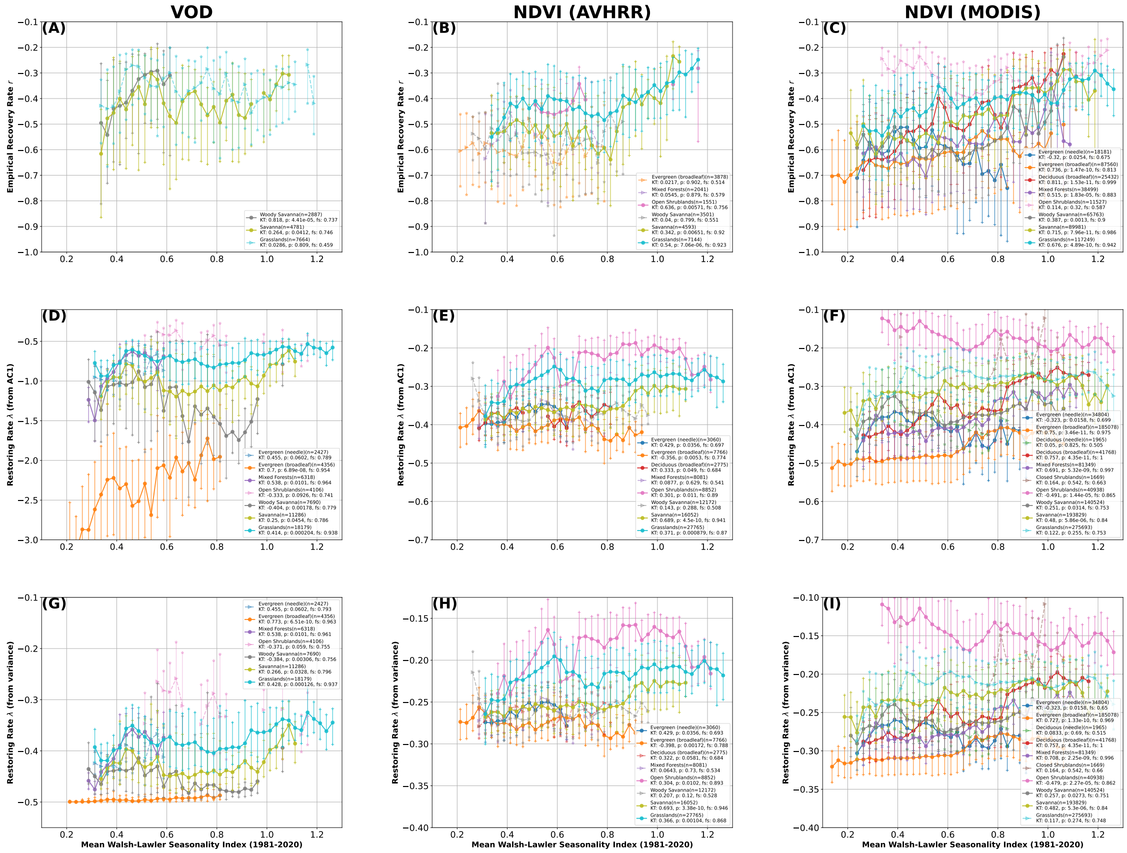

Figure S7: Vegetation resilience as a function of precipitation seasonality in terms of the Walsh-Lawler seasonality index at the global scale, separated by land cover type. Land covers with less than 1000 points or less than 10 bins of at least 50 members are omitted. Vegetation resilience estimated empirically (A,B,C), via the AC1 (D,E,F), and via the variance (G,H,I, Methods) for vegetation optical depth (VOD, left column), GIMMS3g normalized difference vegetation index (NDVI, middle column), and MODIS NDVI (right column). Binned medians shown as solid dots ($p < 0.05$) and transparent arrows ($p > 0.05$), with 25th to 75th percentiles of each bin shown as connected vertical lines capped with hatches. Kendall-Tau coefficients of the series of medians of each bin, their p-values, and the fraction of surrogate series consistent with the uncertainties that have the same sign as the median series (see Methods) are reported in the legend.

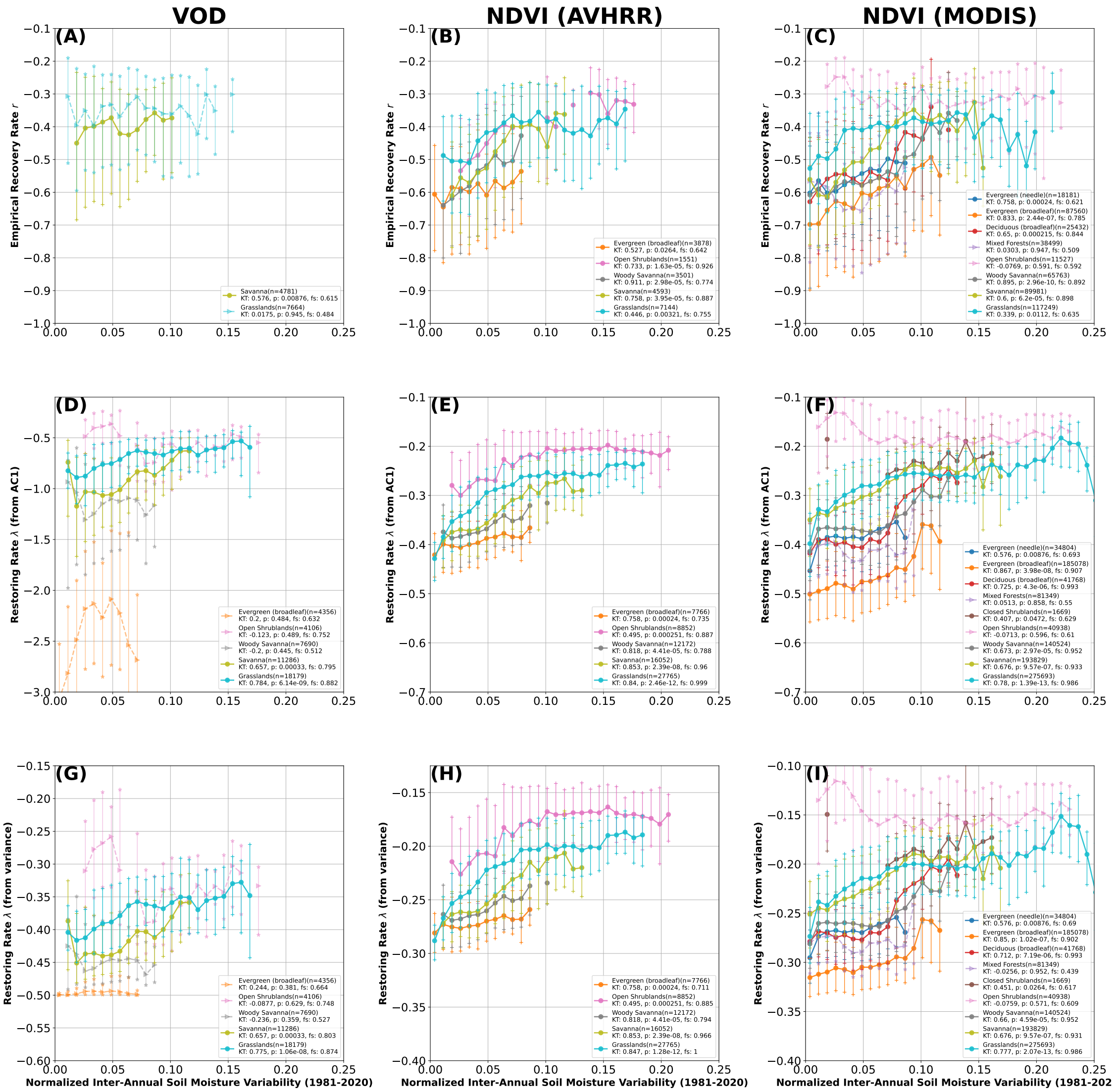

Figure S8: Vegetation resilience as a function of normalized mean annual soil moisture variability (1981-2020, ERA) at the global scale, separated by land cover type. Land covers with less than 1000 points or less than 10 bins of at least 50 members are omitted. Vegetation resilience estimated empirically (A,B,C), via the AC1 (D,E,F), and via the variance (G,H,I, Methods) for vegetation optical depth (VOD, left column), GIMMS3g normalized difference vegetation index (NDVI, middle column), and MODIS NDVI (right column). Binned medians shown as solid dots (p <0.05) and transparent arrows (p >0.05), with 25th to 75th percentiles of each bin shown as connected vertical lines capped with hatches. Kendall-Tau coefficients of the series of medians of each bin, their p-values, and the fraction of surrogate series consistent with the uncertainties that have the same sign as the median series (see Methods) are reported in the legend.

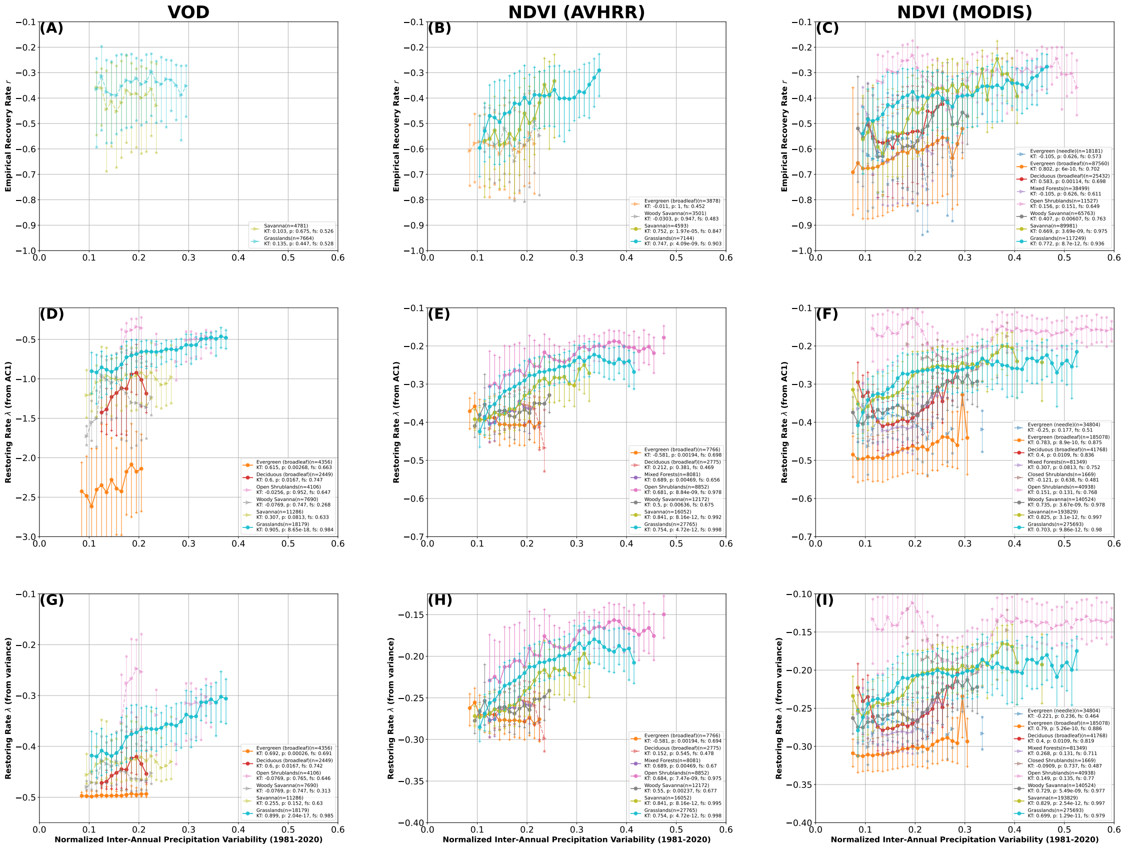

Figure S9: Vegetation resilience as a function of normalized mean annual precipitation variability (1981-2020, ERA) at the global scale, separated by land cover type. Land covers with less than 1000 points or less than 10 bins of at least 50 members are omitted. Vegetation resilience estimated empirically (A,B,C), via the AC1 (D,E,F), and via the variance (G,H,I, Methods) for vegetation optical depth (VOD, left column), GIMMS3g normalized difference vegetation index (NDVI, middle column), and MODIS NDVI (right column). Binned medians shown as solid dots (p <0.05) and transparent arrows (p >0.05), with 25th to 75th percentiles of each bin shown as connected vertical lines capped with hatches. Kendall-Tau coefficients of the series of medians of each bin, their p-values, and the fraction of surrogate series consistent with the uncertainties that have the same sign as the median series (see Methods) are reported in the legend.

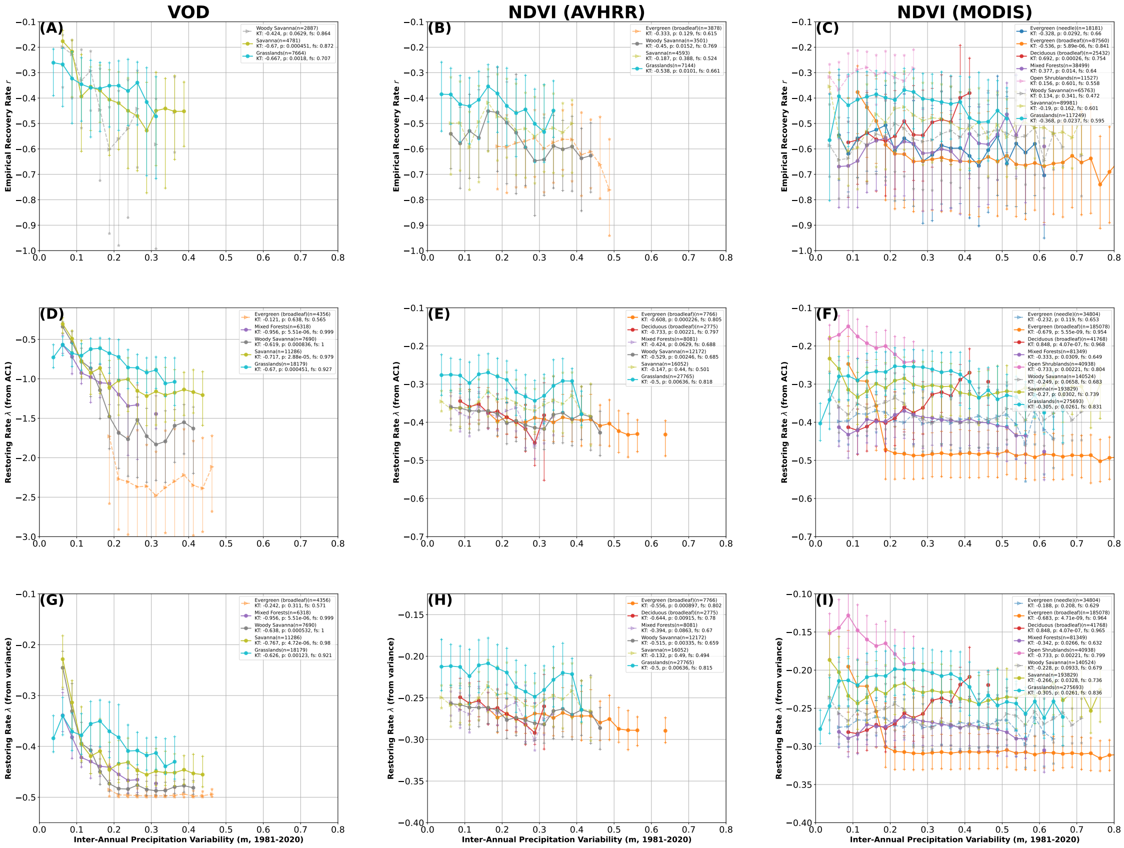

Figure S10: Vegetation resilience as a function of mean annual precipitation standard deviation (1981-2020, ERA) at the global scale, separated by land cover type. Land covers with less than 1000 points or less than 10 bins of at least 50 members are omitted. Vegetation resilience estimated empirically (A,B,C), via the AC1 (D,E,F), and via the variance (G,H,I, Methods) for vegetation optical depth (VOD, left column), GIMMS3g normalized difference vegetation index (NDVI, middle column), and MODIS NDVI (right column). Binned medians shown as solid dots ($p < 0.05$) and transparent arrows ($p > 0.05$), with 25th to 75th percentiles of each bin shown as connected vertical lines capped with hatches. Kendall-Tau coefficients of the series of medians of each bin, their p-values, and the fraction of surrogate series consistent with the uncertainties that have the same sign as the median series (see Methods) are reported in the legend.

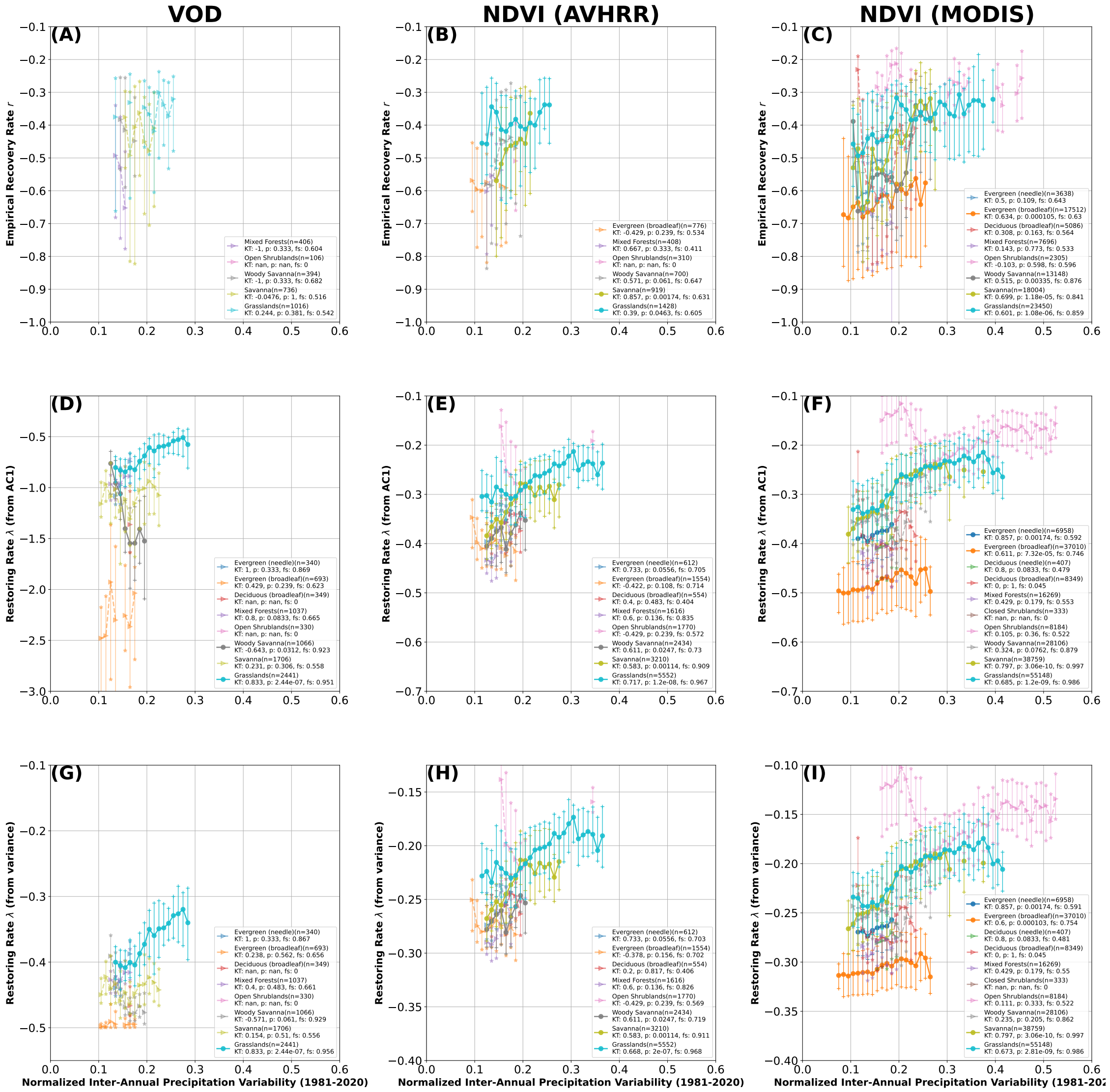

Figure S11: Vegetation resilience as a function of normalized mean annual precipitation variability (1981-2020, ERA) at the global scale, separated by land cover type. Land covers with less than 1000 points or less than 10 bins of at least 50 members are omitted. Vegetation resilience estimated empirically (A,B,C), via the AC1 (D,E,F), and via the variance (G,H,I, Methods) for vegetation optical depth (VOD, left column), GIMMS3g normalized difference vegetation index (NDVI, middle column), and MODIS NDVI (right column). Binned medians shown as solid dots (p <0.05) and transparent arrows (p >0.05), with 25th to 75th percentiles of each bin shown as connected vertical lines capped with hatches. Kendall-Tau coefficients of the series of medians of each bin, their p-values, and the fraction of surrogate series consistent with the uncertainties that have the same sign as the median series (see Methods) are reported in the legend. Only data around the median of precipitation (40th to 60th percentile mean annual precipitation) by land cover type shown.

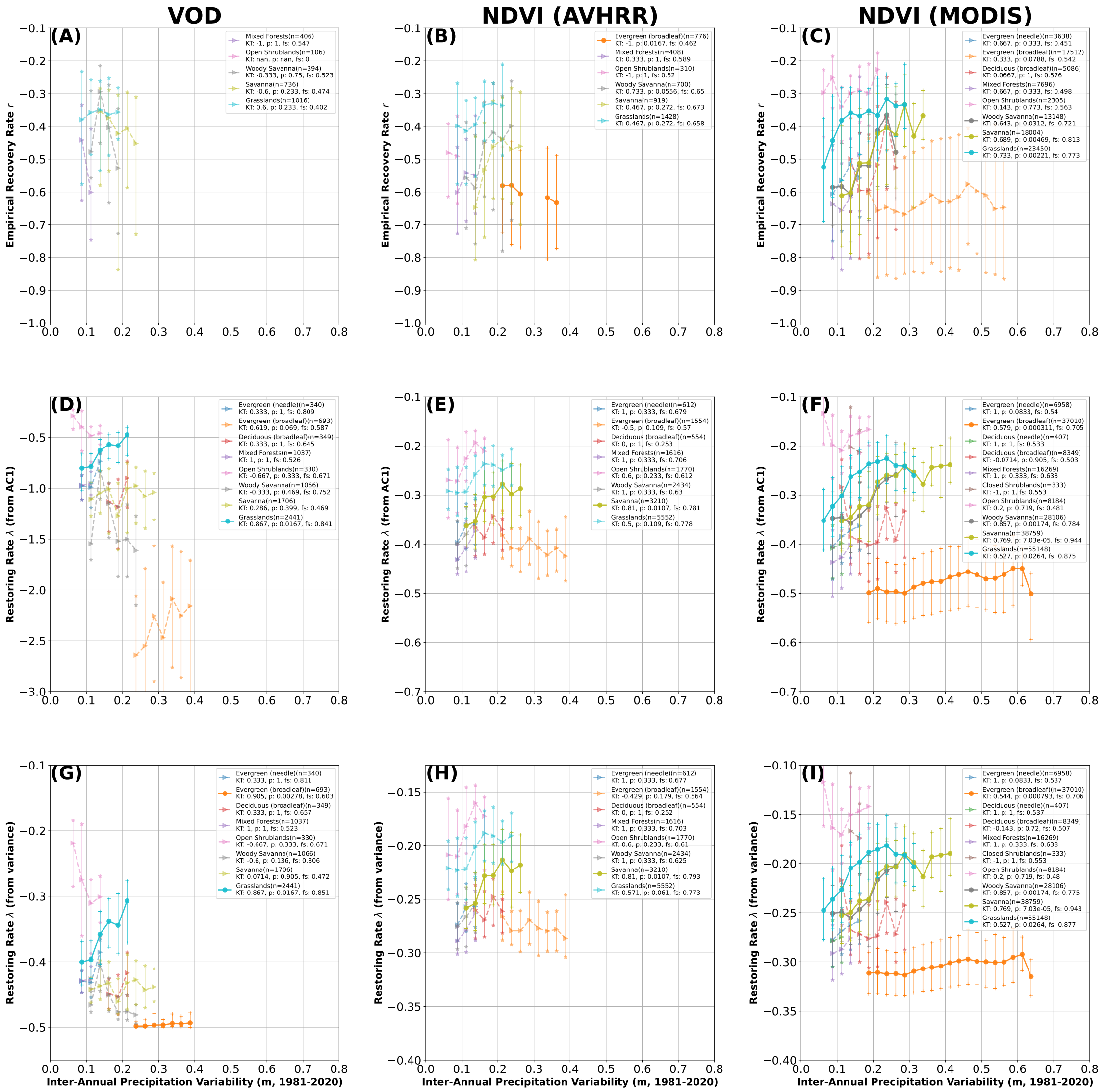

Figure S12: Vegetation resilience as a function of mean annual precipitation standard deviation (1981-2020, ERA) at the global scale, separated by land cover type. Land covers with less than 1000 points or less than 10 bins of at least 50 members are omitted. Vegetation resilience estimated empirically (A,B,C), via the AC1 (D,E,F), and via the variance (G,H,I, Methods) for vegetation optical depth (VOD, left column), GIMMS3g normalized difference vegetation index (NDVI, middle column), and MODIS NDVI (right column). Binned medians shown as solid dots (p <0.05) and transparent arrows (p >0.05), with 25th to 75th percentiles of each bin shown as connected vertical lines capped with hatches. Kendall-Tau coefficients of the series of medians of each bin, their p-values, and the fraction of surrogate series consistent with the uncertainties that have the same sign as the median series (see Methods) are reported in the legend. Only data around the median of precipitation (40th to 60th percentile mean annual precipitation) by land cover type shown.

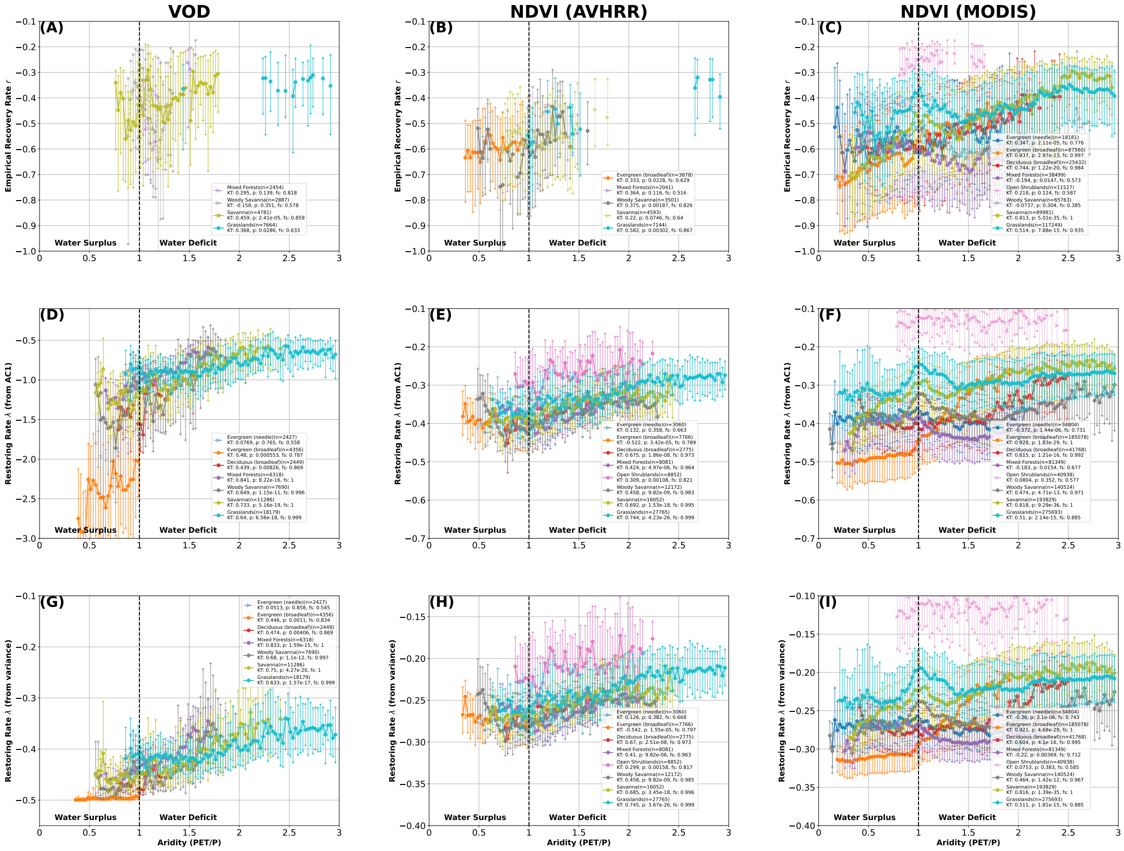

Figure S13: Vegetation resilience as a function of aridity at the global scale, separated by land cover type. Land covers with less than 1000 points or less than 10 bins of at least 50 members are omitted. Vegetation resilience estimated empirically (A,B,C), via the AC1 (D,E,F), and via the variance (G,H,I, Methods) for vegetation optical depth (VOD, left column), GIMMS3g normalized difference vegetation index (NDVI, middle column), and MODIS NDVI (right column). Binned medians shown as solid dots (p <0.05) and transparent arrows (p >0.05), with 25th to 75th percentiles of each bin shown as connected vertical lines capped with hatches. Kendall-Tau coefficients of the series of medians of each bin, their p-values, and the fraction of surrogate series consistent with the uncertainties that have the same sign as the median series (see Methods) are reported in the legend. Smaller (0.025) bin sizes than Figure 3.

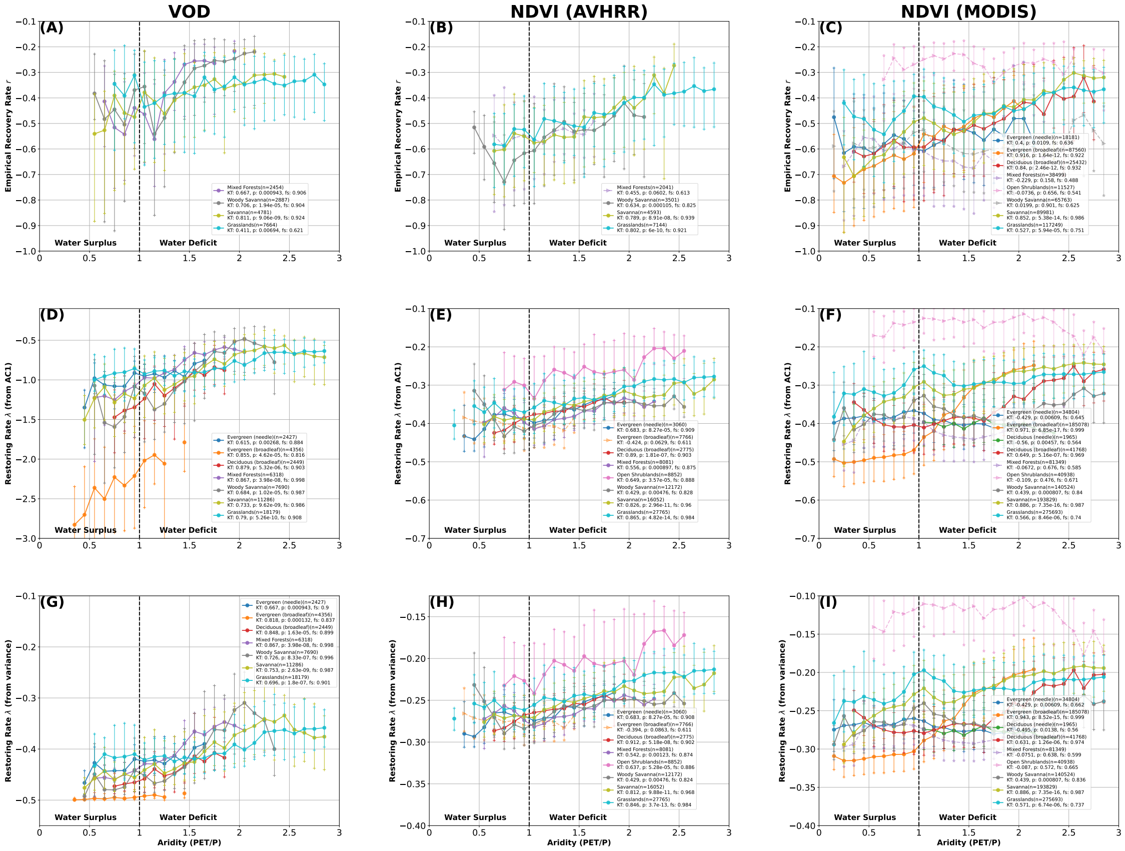

Figure S14: Vegetation resilience as a function of aridity at the global scale, separated by land cover type. Land covers with less than 1000 points or less than 10 bins of at least 50 members are omitted. Vegetation resilience estimated empirically (A,B,C), via the AC1 (D,E,F), and via the variance (G,H,I, Methods) for vegetation optical depth (VOD, left column), GIMMS3g normalized difference vegetation index (NDVI, middle column), and MODIS NDVI (right column). Binned medians shown as solid dots (p <0.05) and transparent arrows (p >0.05), with 25th to 75th percentiles of each bin shown as connected vertical lines capped with hatches. Kendall-Tau coefficients of the series of medians of each bin, their p-values, and the fraction of surrogate series consistent with the uncertainties that have the same sign as the median series (see Methods) are reported in the legend. Larger (0.1) bin sizes than Figure 3.
