# Supplementary material for: Global vegetation resilience linked to water availability and variability
Source: Nat Commun. 2023 Jan 30;14:498. doi: 10.1038/s41467-023-36207-7 (PMC9886942; doi:10.1038/s41467-023-36207-7)
Supplement: Supplementary file 3 — Reporting Summary [file 41467_2023_36207_MOESM3_ESM.pdf]

nature portfolio

Corresponding author(s): Smith, Taylor

Last updated by author(s): Jan 5, 2023

Reporting Summary

Nature Portfolio wishes to improve the reproducibility of the work that we publish. This form provides structure for consistency and transparency in reporting. For further information on Nature Portfolio policies, see our Editorial Policies and the Editorial Policy Checklist.

Statistics

For all statistical analyses, confirm that the following items are present in the figure legend, table legend, main text, or Methods section.

n/a	Confirmed	
☐	☒	The exact sample size (*n*) for each experimental group/condition, given as a discrete number and unit of measurement
☒	☐	A statement on whether measurements were taken from distinct samples or whether the same sample was measured repeatedly
☐	☒	The statistical test(s) used AND whether they are one- or two-sided<br>*Only common tests should be described solely by name; describe more complex techniques in the Methods section.*
☐	☒	A description of all covariates tested
☒	☐	A description of any assumptions or corrections, such as tests of normality and adjustment for multiple comparisons
☐	☒	A full description of the statistical parameters including central tendency (e.g. means) or other basic estimates (e.g. regression coefficient) AND variation (e.g. standard deviation) or associated estimates of uncertainty (e.g. confidence intervals)
☐	☒	For null hypothesis testing, the test statistic (e.g. *F*, *t*, *r*) with confidence intervals, effect sizes, degrees of freedom and *P* value noted<br>*Give P values as exact values whenever suitable.*
☒	☐	For Bayesian analysis, information on the choice of priors and Markov chain Monte Carlo settings
☒	☐	For hierarchical and complex designs, identification of the appropriate level for tests and full reporting of outcomes
☒	☐	Estimates of effect sizes (e.g. Cohen's *d*, Pearson's *r*), indicating how they were calculated

Our web collection on statistics for biologists contains articles on many of the points above.

Software and code

Policy information about availability of computer code

Data collection	Data assimilation and processing was done using Python [v. 3.9.13], based on publicly available data.
Data analysis	Data analysis was performed using the Python [v. 3.9.13] language. Analysis codes can be found on Zenodo: 10.5281/zenodo.7436669

For manuscripts utilizing custom algorithms or software that are central to the research but not yet described in published literature, software must be made available to editors and reviewers. We strongly encourage code deposition in a community repository (e.g. GitHub). See the Nature Portfolio guidelines for submitting code & software for further information.

Data

Policy information about availability of data

All manuscripts must include a data availability statement. This statement should provide the following information, where applicable:

- Accession codes, unique identifiers, or web links for publicly available datasets
- A description of any restrictions on data availability
- For clinical datasets or third party data, please ensure that the statement adheres to our policy

The Kendall-Tau statistics generated in this study have been deposited on Zenodo at 10.5281/zenodo.7436669. The raw environmental and satellite data used in this study are publicly available. Direct links to the data sets can be found at: GIMMS NDVI: https://www.cen.uni-hamburg.de/en/icdc/data/land/gimms-ndvi3g.html, VOD: https://zenodo.org/record/2575599, MODIS Land Cover: https://lpdaac.usgs.gov/products/mcd12q1v006/, MODIS NDVI: https://lpdaac.usgs.gov/products/mod13c1v006/, WorldCLIM Aridity: https://figshare.com/articles/dataset/Global_Aridity_Index_and_Potential_Evapotranspiration_ET0_Climate_Database_v2/7504448/4, ERA5 Climate Data: https://www.ecmwf.int/en/forecasts/

datasets/reanalysis-datasets/era5.

Human research participants

Policy information about studies involving human research participants and Sex and Gender in Research.

Reporting on sex and gender	N/A
Population characteristics	N/A
Recruitment	N/A
Ethics oversight	N/A

Note that full information on the approval of the study protocol must also be provided in the manuscript.

Field-specific reporting

Please select the one below that is the best fit for your research. If you are not sure, read the appropriate sections before making your selection.

☐ Life sciences ☐ Behavioural & social sciences ☒ Ecological, evolutionary & environmental sciences

For a reference copy of the document with all sections, see nature.com/documents/nr-reporting-summary-flat.pdf

Ecological, evolutionary & environmental sciences study design

All studies must disclose on these points even when the disclosure is negative.

Study description	This study investigated the relationship between water availability and vegetation resilience at a global scale. It used publicly available satellite data to test the hypothesis that vegetation in more favorable conditions (e.g., with more or more consistent water) is more resilient to shocks.
Research sample	We sampled all vegetated areas which had not been significantly influence by humans (e.g., farms, urban areas) and had not changed land-cover types (e.g., from Forest to Savanna) over the study period. We excluded human-influenced areas to study only natural changes in ecosystems (i.e. those not caused by e.g. agriculture). This analysis was global, and relied on multiple satellite data sets, all of which are publicly available:<br><br>GIMMS NDVI: https://www.cen.uni-hamburg.de/en/icdc/data/land/gimms-ndvi3g.html,<br>VOD: https://zenodo.org/record/2575599,<br>MODIS Land Cover: https://lpdaac.usgs.gov/products/mcd12q1v006/,<br>MODIS NDVI: https://lpdaac.usgs.gov/products/mod13c1v006/,<br>WorldCLIM Aridity: https://figshare.com/articles/dataset/<br>Global_Aridity_Index_and_Potential_Evapotranspiration_ET0_Climate_Database_v2/7504448/4,<br>ERA5 Climate Data: https://www.ecmwf.int/en/forecasts/datasets/reanalysis-datasets/era5.
Sampling strategy	We used all available satellite measurements that fulfilled the above sampling conditions.
Data collection	Data was provided by NASA (MODIS, AVHRR) and through the published VODCA data record, based on various passive microwave satellite data sets. We did not perform data collection, but relied upon previously published data.
Timing and spatial scale	Data generally ranged from the late 80s till 2020, depending on data source. Sampling was bi-weekly. Spatial scale was between 5 and 25 km.
Data exclusions	Data was excluded if there was significant human land cover, as we could no longer look for relationships in natural vegetation in this case. E.g., farms do not follow a natural annual water cycle, but rather respond to human-induced watering changes.
Reproducibility	All codes needed to reproduce our results are available in Zenodo. All data is open source.
Randomization	Data was divided primarily by land-cover type. This is a necessity when comparing vegetation with different basic functions -- the inherent speed of plant growth varies from place to place and by ecosystem.
Blinding	Blinding was not relevant to our study -- we did not collect the data.

Did the study involve field work? ☐ Yes ☒ No

Reporting for specific materials, systems and methods

We require information from authors about some types of materials, experimental systems and methods used in many studies. Here, indicate whether each material, system or method listed is relevant to your study. If you are not sure if a list item applies to your research, read the appropriate section before selecting a response.

Materials & experimental systems

n/a	Involved in the study
☒	☐ Antibodies
☒	☐ Eukaryotic cell lines
☒	☐ Palaeontology and archaeology
☒	☐ Animals and other organisms
☒	☐ Clinical data
☒	☐ Dual use research of concern

Methods

n/a	Involved in the study
☒	☐ ChIP-seq
☒	☐ Flow cytometry
☒	☐ MRI-based neuroimaging
